# Facile Tumor Spheroids Formation in Large Quantity with Controllable Size and High Uniformity

**DOI:** 10.1038/s41598-018-25203-3

**Published:** 2018-05-01

**Authors:** Wentao Shi, Jean Kwon, Yongyang Huang, Jifu Tan, Christopher G. Uhl, Ran He, Chao Zhou, Yaling Liu

**Affiliations:** 1 Department of Bioengineering, Lehigh University, Bethlehem, Pennsylvania 18015 USA; 2 Department of Electrical and Computer Engineering, Lehigh University, Bethlehem, Pennsylvania 18015 USA; 3 Department of Mechanical Engineering, Northern Illinois University, DeKalb, IL 60115 USA; 4 Department of Mechanical Engineering and Mechanics, Lehigh University, Bethlehem, Pennsylvania 18015 USA

## Abstract

A facile method for generation of tumor spheroids in large quantity with controllable size and high uniformity is presented. HCT-116 cells are used as a model cell line. Individual tumor cells are sparsely seeded onto petri-dishes. After a few days of growth, separated cellular islets are formed and then detached by dispase while maintaining their sheet shape. These detached cell sheets are transferred to dispase-doped media under orbital shaking conditions. Assisted by the shear flow under shaking and inhibition of cell-to-extracellular matrix junctions by dispase, the cell sheets curl up and eventually tumor spheroids are formed. The average size of the spheroids can be controlled by tuning the cell sheet culturing period and spheroid shaking period. The uniformity can be controlled by a set of sieves which were home-made using stainless steel meshes. Since this method is based on simple petri-dish cell culturing and shaking, it is rather facile for forming tumor spheroids with no theoretical quantity limit. This method has been used to form HeLa, A431 and U87 MG tumor spheroids and application of the formed tumor spheroids in drug screening is also demonstrated. The viability, 3D structure, and necrosis of the spheroids are characterized.

## Introduction

Three-dimensional (3D) cell culture systems have shown many important advantages over two-dimensional (2D) cell culture systems, 3D models more accurately mimic the complex *in vivo* microenvironment and produce cellular behavior more close to natural conditions^1–4^. In the area of cancer research, 3D multicellular tumor spheroids (MCTS) have attracted more and more attention for their potential applications in anti-cancer drug screening and *in vitro* tumor studies over 2D tumor cell cultures^5–7^. Current standard MCTS formation methods include the Liquid-Overlay method^8,9^ and the Hanging-Drop method^3,10^, both of which can produce size-controllable and highly uniform MCTS. However, since only a single spheroid can be formed in one well or one droplet, the amount of MCTS these two methods can produce is rather limited, let alone the tedious labor required for MCTS preparation. Other methods such as Magnetic Levitation^11,12^ and the NASA Bioreactor^13,14^ may produce large quantities of MCTS, but the uniformity is a problem and the ratio of spherical spheroids is rather low^15^. Uniformity of MCTS determines the feasibility of their application in drug screening industry. What is more, the Magnetic Levitation method recruits magnetic particles and the NASA Bioreactor method needs an expensive bioreactor. Therefore, a better MCTS formation method is required to benefit the research, development, and clinical community by facilitating the process to produce replicate spheroids of uniform size and mass quantities while being low cost.

Herein, a novel method that can readily produce tumor spheroids with controllable size in large quantity and high uniformity is proposed. By using a special protein dispase, which only cleaves cell-Extracellular matrix (ECM) junctions, sparsely distributed tumor cell sheets are separated from the culturing surface. By shaking these separated cell sheets within dispase-doped media, which prevents stacking of cell sheets, robust tumor spheroids are formed. The spheroids size can be controlled by tuning the cell sheet culturing period and spheroid shaking period. The uniformity can be controlled by a set of sieves which were home-made by stainless steel meshes. Basic characterization was performed to study the viability, 3D structure and necrosis of the spheroids, and their application in drug screening is also demonstrated.

## Results

### Formation Process

**Figure 1:**
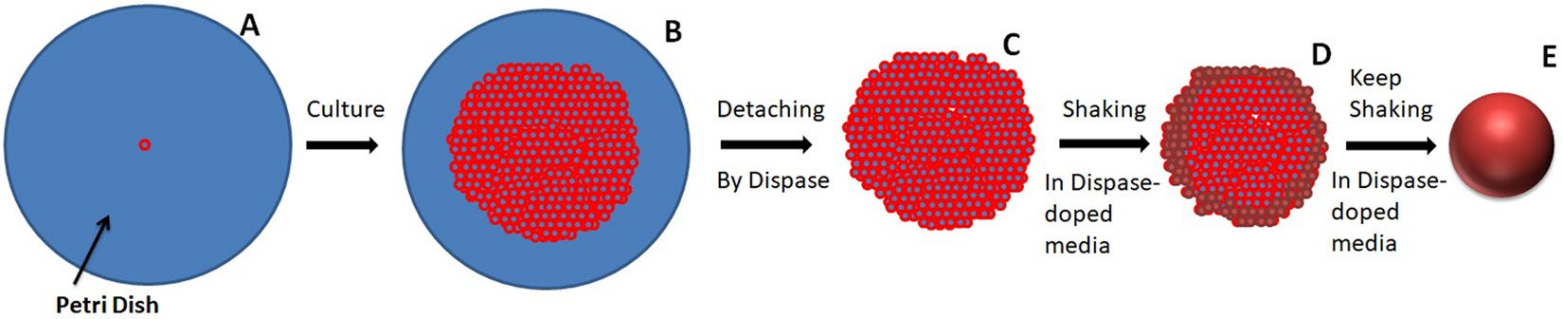
Cartoon illustration of cellular spheroid formation. Single cells are seeded onto petri dishes (**A**), and after a few days of growth, cell sheets form (**B**). Cell sheets can be detached from the petri dishes by dispase and the sheets can be maintained (**C**). Shaking the cell sheets in dispase-doped media allows their edges fold inward (**D**) and eventually the cell sheets become spheroids (**E**).

**Figure 2:**
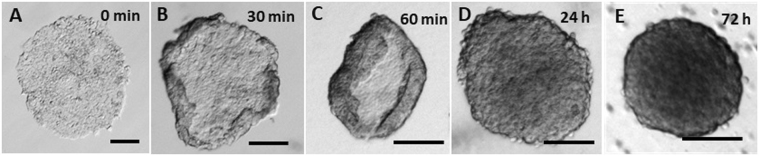
Contrast microscope images of HCT-116 tumor spheroids formation process. (**A**) HCT-116 cell sheet (Sheet growth = 10 days) on petri dish and (**B**–**E**) cell sheet was detached by dispase and kept orbital-shaking at 30 rph with dispase doped medium (1/6) for different time. All scale bars are 200 µm.

A cartoon illustration of MCTS formation can be found in Fig. 1. Single cells were first seeded onto tissue-culture treated petri dishes. To ensure each cell had sufficient room to grow as a separate cell sheet, the initial cell density/concentration was kept low. The low cell density resulted in sparsely distributed single HCT116 cells, which grew slowly in the first several days after sub-culturing; the cells required at least 6 days to form cell sheets of desirable sizes on the petri dishes^16^. 6 days after the cell seeding, the cell sheets began to grow vigorously, covering as much as an area of 2 mm^2^ by day 13. The released cell sheets were then orbitally shaken with dispase doped media, and the edges began to curl, as shown in Fig. 2B. Interestingly, as far as we observed, all the curling took place on a same side. During shaking, the curling proceeded towards the center until a closed shape was formed within 24 h. Usually after 72 h of shaking with dispase-doped media, a spherical shape formed. Control experiments were performed, and the results can be found in Fig. S1. Briefly, shaking cell sheets in media without dispase would result in stacked cell sheets, as shown in Fig. S1A; stopping the shaking process for 24 h would result in spheroid aggregation, as shown in Fig. S1B; shaking single cell suspension other than cell sheets would also result in aggregations. Moreover, when the sheet culturing time was too long (more than 14 days), thick cell sheets would form, and shaking these cell sheets in dispase doped media would result in thick, round-shaped cell sheets instead of spheroids, as shown in Fig. S1C. After spheroids of a significantly large size (usually average diameter > 200 μm) were obtained, fresh dispase-free media was changed by using a home-made bio-sieve. The cytotoxicity of dispase is shown in Fig. S2, and it was found that dispase has negligible negative effects on cell and spheroid growth, as well as cell-cell junction proteins such as E-cadherin (Fig. S2E). Besides HCT-116, the tumor spheroid formation method could be also applied to other tumor cell lines. Microscopic images of spheroids made of A431, HeLa cells and U87 MG are found in Fig. S4.

### Size Control

**Figure 3:**
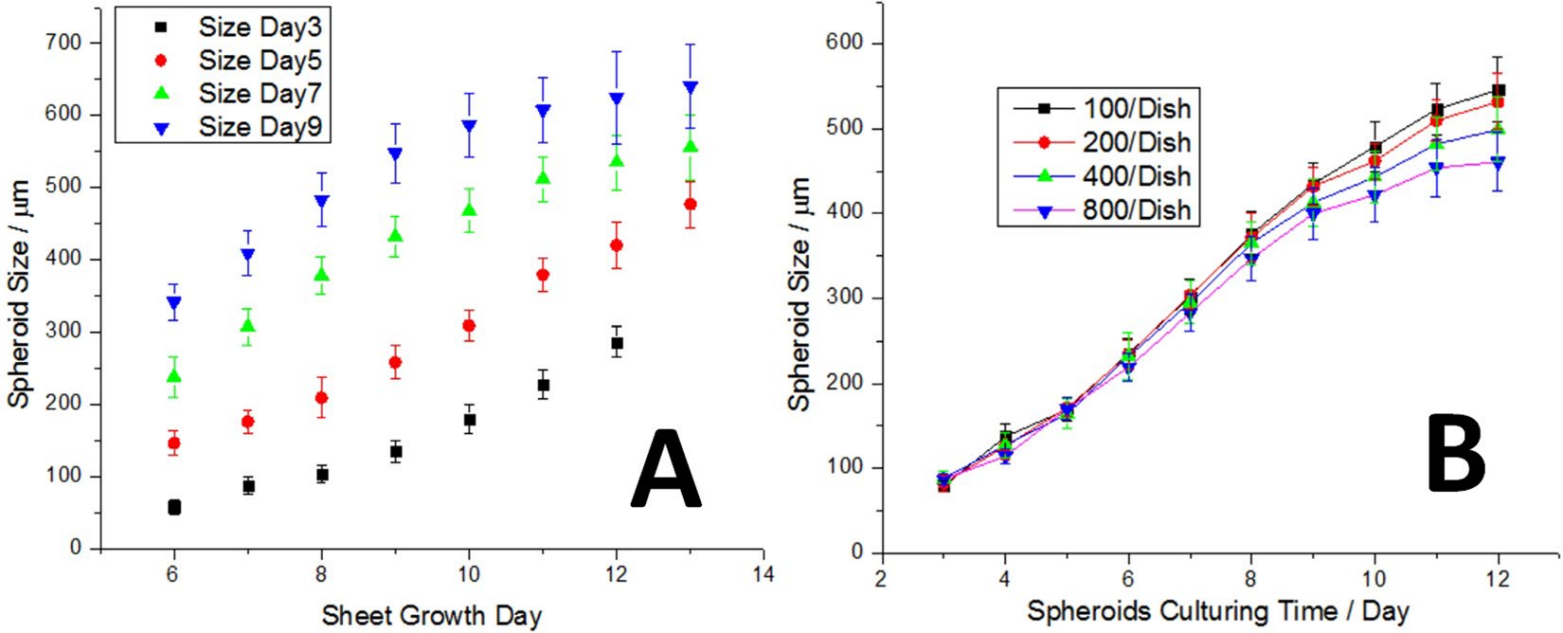
Size control of HCT-116 tumor spheroids. (**A**) Size comparison of spheroids from cell sheets cultured on petri dishes for different times (6 days to 13 days). Spheroid sizes were recorded on different shaking days (black, red, green and blue dots are recorded on shaking day 3, 5, 7, and 9, respectively). (**B**) Size comparison of spheroids from cell sheets that are from different initial cell seeding densities. Spheroid sizes were recorded for 12 days. The tumor spheroids were not perfectly spherical, and the estimation of mean spheroid diameter (D) was suggested by^28^, which was simply calculated as the square root of two orthogonal diameters (d1 and d2) of the spheroids product of longest axis and shortest axis: $D=\sqrt{{d}_{1}{d}_{2}}$.

To obtain certain sizes of spheroids, the most important thing is to allow most of the spheroids grow to such size. A few factors will influence the size of spheroids: the time of cell sheet culturing, the time of spheroid shaking, the initial cell seeding density, and the nutrition supply. The spheroids size changes from different cell sheet growth time were recorded for up to 9 days, and the results were shown in Fig. 3A. Obviously, the larger the original cell sheet, the bigger spheroid that would be obtained from the same cell sheet; and the longer the spheroids were cultured in shaken media, the bigger they would be. Larger spheroids tended to grow more slowly, as shown by the decreased slope in Day 7 and 9. The initial cell seeding density was another factor that influenced spheroid size. Spheroid size from cell sheets grown for 7 days of four different initial cell seeding densities were recorded and results are shown in Fig. 3B. Spheroids from higher cell density dishes were significantly smaller than the ones from lower cell density dishes.

### Size Uniformization

**Figure 4:**
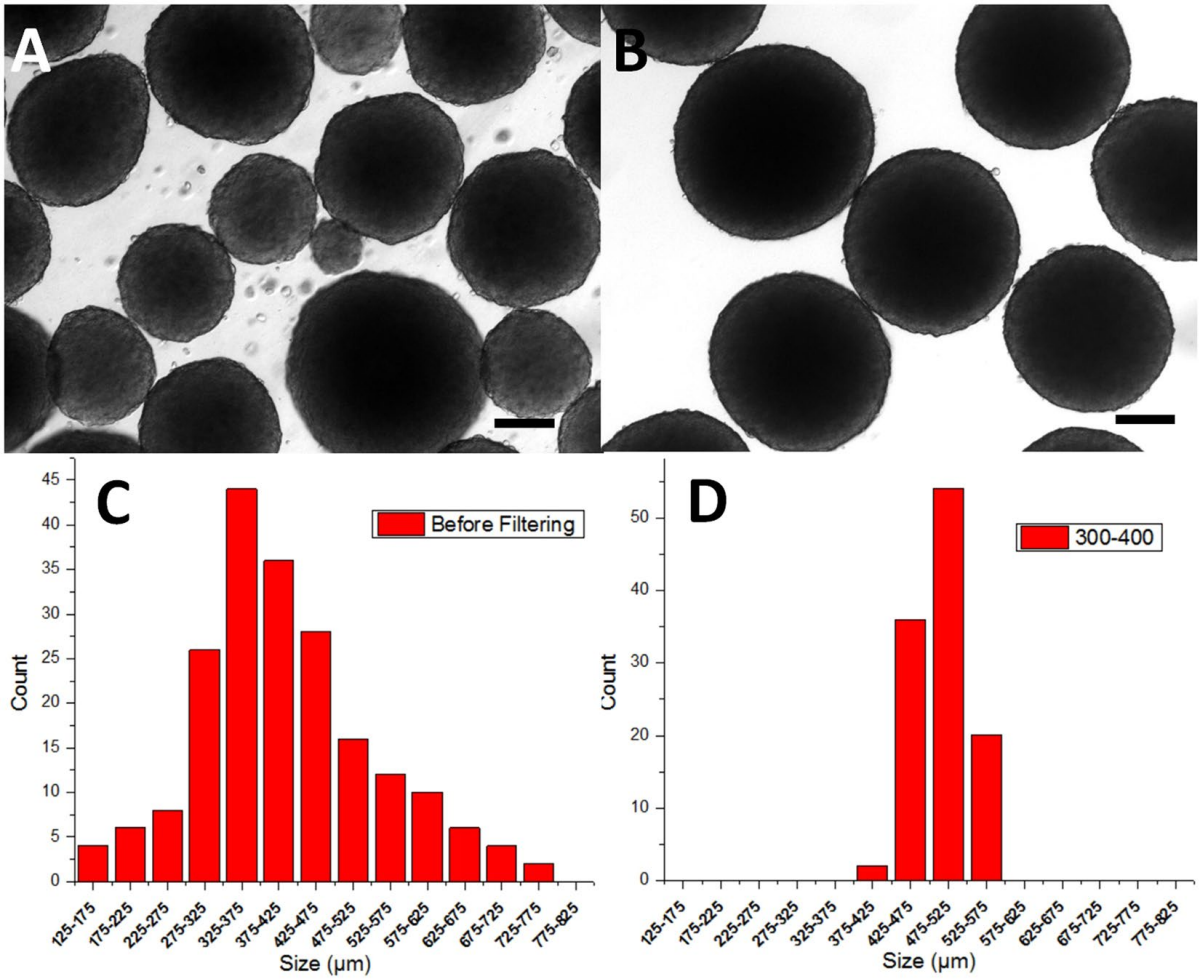
Tumor spheroids size uniformization. (**A**) A typical image showing the wide size-distribution of the tumor spheroids, taken at shaking day 7 (detached at sheet-growth day 9). Tumor spheroids from the same batch as (**A**) filtered from 300–400 µm sieves (**B**). (**C** and **D**) Tumor spheroid size distribution before and after filtration, corresponding to (**A** and **B**). Each spheroid count was counted and calculated from 10–30 images taken at certain size distributions. All scale bars are 200 µm.

To uniformize the size of spheroids, a set of home-made tumor sieves were used, as shown in Fig. S5. Stainless steel meshes were easily found from many sources, and the mesh size could be varied over a rather wide range, so spheroids with different sizes could be uniformly obtained. For example, as shown in Fig. 4B, spheroids filtered with 300–400 μm sieves in the same sample had a rather small variance in size between 370 and 570 μm (Fig. 4D). The size distribution of spheroids from a single batch through a full spectrum between 149 μm and 600 μm can be found in Fig. S6. Obviously, the diameter of filtered spheroids was larger than the mesh size of the larger sieve used for filtration, and the size distribution is significantly narrowed because of the filtration. To obtain even more uniform spheroids, increments between adjacent sizes within 20–30 μm can be found commercially, which would allow for the uniformity to be dramatically increased. For example, the upper image of Fig. S7 showed a low magnification image of highly packed spheroids filtered by 120 and 144 µm sieves, with equivalent diameter (range, mean ± SD, CV, n) of (172–241 µm, 201 ± 13 µm, 6.35, and 460) respectively. The spheroids can be applied to 96-well or 384-well straightforwardly for drug testing or other applications. Currently, we can only pipette each spheroid into the wells individually. 96 spheroids were randomly picked from the 460 spheroids and placed into a 96 well plate. The bright field images of each spheroid in the well plate were show in the lower figure of Fig. S7.

### Characterization

**Figure 5:**
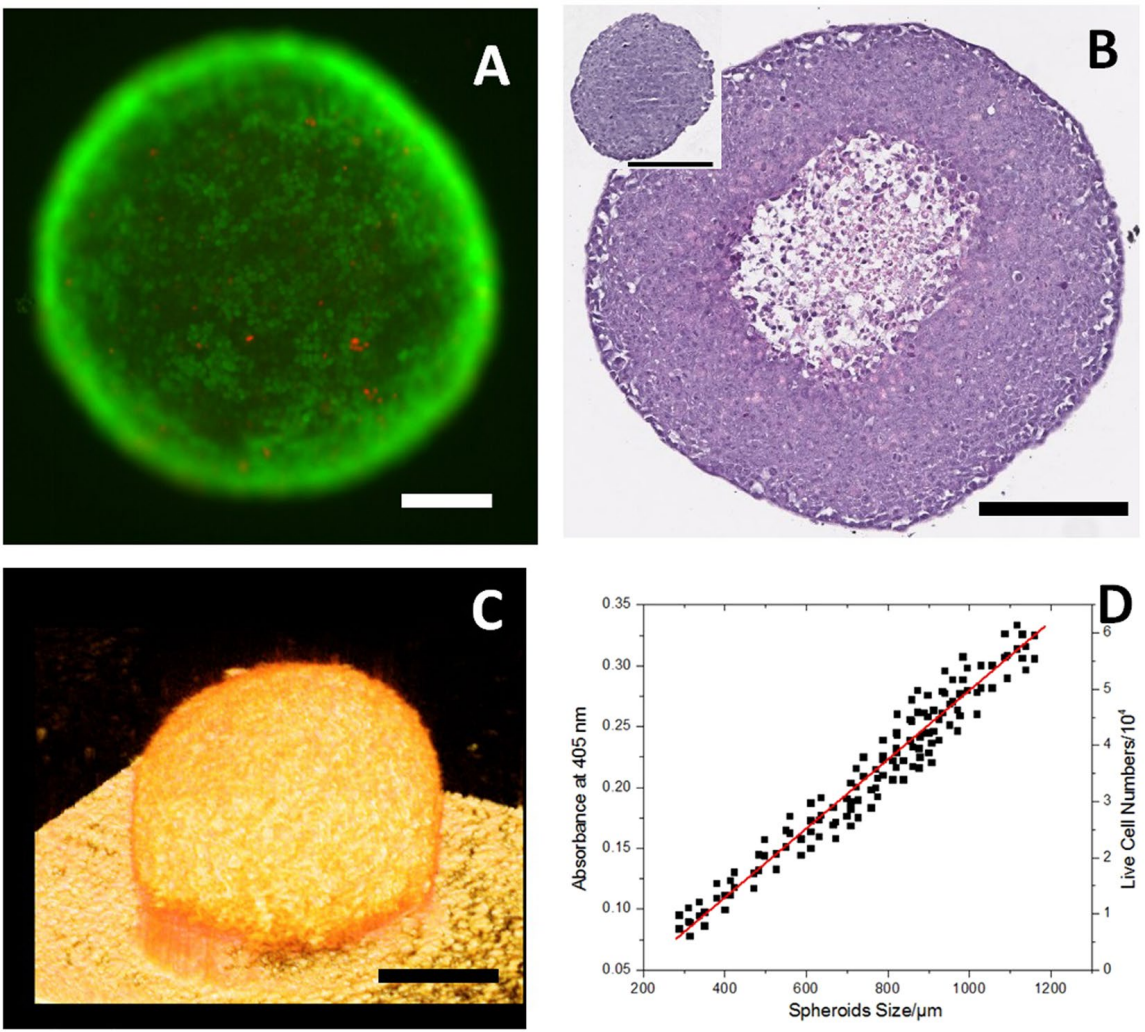
Basic tumor spheroid characteristics. (**A**) Representative dead/live staining on a HCT116 spheroid. (**B**) Representative microscopic images of H&E stained 5 µm paraffin embedded sections of HCT116 spheroids with (the bigger one) and without (the smaller one) central secondary necrosis. (**C**) 3D OCT images of a HCT116 spheroid with 558 µm diameter showing that spherical shape is maintained for the large spheroid. (**D**) The linear relationship between spheroid size and its live cell numbers. (**D**) Was plotted with about 130 spheroids with size ranging from 300 µm to 1200 µm. Scale bars = 200 μm.

Dead and live staining was used to examine the viability of tumor spheroids. Figure 5A is a fluorescence microscope image of a spheroid with 875 μm diameter. Live cells emitting green occupied almost the entire surface, with a few dead cells emitting red. This indicated that spheroids obtained by this method were robust. Confocal fluorescence microscopy was also used to examine deeper into the spheroids, but no desirable results were obtained, maybe due to the low permeability of the dyes. The inner structure could be investigated by histology. As shown in Fig. 5B, spheroids with smaller diameter (~258 µm) did not exhibit central secondary necrosis, while the larger ones (~580 µm) developed central cell death and formed necrosis. The thickness of viable cell shell for the spheroids is about 200 µm, which supports the conclusions made by plenty of publications that the maximum thickness of viable cell shell for spheroids is about 150–200 µm^17^. The 3D structure information was examined by Optical Coherence Tomography (OCT). As shown in Figs 5C and S8, spheroids with both smaller and larger diameters could retain a spherical shape. Viable cell numbers per spheroid is another important parameter of tumor spheroids, which was a prerequisite for the evaluation of commercial cell viability/cytotoxicity assays. APH assay was performed on different sizes of spheroids, and interestingly a linear relationship was found between the spheroid diameter and viable cell numbers in the spheroids, which was consistent with findings in literature^18^. The linear calibration of viable cell numbers to absorbance at 405 nm in well plates can be found in Figs 5D and S9.

### Drug Test

**Figure 6:**
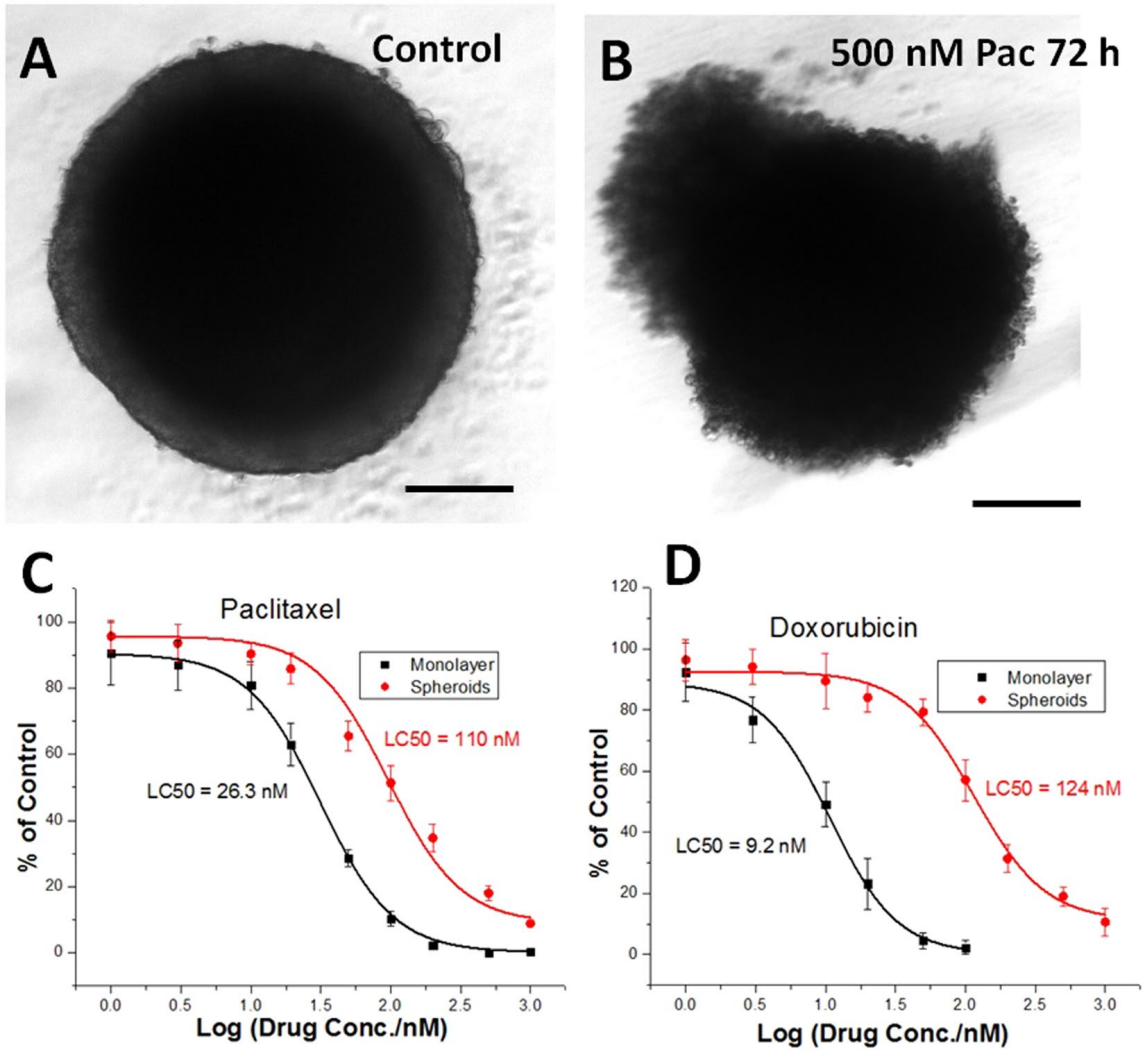
The drug effect on HCT116 tumor spheroids by APH assay. Similar size spheroids (diameter ~500 µm) were treated with different concentrations of Paclitaxel and Doxorubicin for 72 h, and (**A**) shows a typical controlled spheroid, while (**B**) shows a spheroid that was treated by 500 nM Paclitaxel for 72 h. More detailed images can be found in Fig. S10. Drug effects of Paclitaxel (**C**) and Doxorubicin (**D**) in spheroids cultures, with comparison to monolayer cell cultures. Dose-response curves were plotted exponentially. Scale bars = 200 µm.

To examine the drug-screening applications of this method, Paclitaxel and Doxorubicin were used to test the drug-resistance of tumor spheroids and a comparison to monolayer cell culture was made. Both of the two drugs showed excellent cytotoxicity to HCT116 cells^19,20^, even though the mechanisms are different. Robust single tumor spheroids about 500 µm in diameter obtained from 322–352 µm sieves, were seeded per well. Paclitaxel and Doxorubicin in different concentrations of doped media were used to treat the spheroids for 72 h. As shown in Fig. 4B, after 72 h treatment, the 500 nM Pac treated spheroid was broken and obviously the volume was not as large as the control (Fig. 6A), showing remarkable cell loss. APH assay was used to determine the drug effect of the two drugs on the tumor spheroids. As shown in Fig. 6C,D, tumor spheroids showed a higher drug-resistance than monolayer cell cultures. Comparing the two drugs, paclitaxel showed less difference between tumor spheroids and monolayer cell culture. For example, APH activity in monolayer was reduced about 70% with 72 h of 50 nM Pac treatment, compared to about 35% in spheroids, while with same time and same concentration of Dox treatment, APH activity in monolayer was reduced about 95%, compared to just about 20% in spheroids. This indicated that HCT116 spheroids had higher drug-resistance to Dox than monolayer cell culture, compared to Pac. The half maximal inhibitory concentration (IC50) values in spheroids culture were 124 ± 20 nM for Dox and 110 ± 19 nM for Pac, and IC50 values in monolayer cultures were 9.22 ± 4.25 nM for Dox and 26.3 ± 12.5 nM for Pac. A set of 3D rendered OCT images of drug treated spheroids were shown in Fig. S11. The initial size of spheroids were about 250 µm. The spheroid which did not receive any drug treatment showed an intact spherical shape and grew to about 420 µm diameter in 72 h, while the spheroid treated with 500 nM Pac was broken, with dead cells burst out of the spheroid. Although no such dead cell burst was found in Fig. S11C, which was a spheroid treated with 500 nM Dox for 72 h, the integrity of the spheroid was not as intact as the untreated one, and the volume was obviously smaller than the spheroid in Fig. S11A. Actually, since the handling was careful, the two drug treated spheroids did not experience severe shaking and high shear rate. One time of media change or pipetting would break the drug treated spheroids, which was another proof that the structure of such spheroids was rather loose. On the contrary, the untreated spheroid would stay intact no matter how many times of handling and transferring occur. Here, we mainly demonstrate the tumor spheroid formation method, and the mechanism of the difference between drugs still needs further studies.

## Discussion

Dispase is a low toxicity protease^21^ which only cleaves fibronectin, collagen IV, and other cell-ECM connections, while barely affecting cell-cell junctions such as adherence junctions. As such, dispase treatment on tumor cell sheets is capable of lifting intact sheets off of petri dishes without breaking the sheets apart. As the results of Dot plot assay of E-cadherin in Fig. S2 suggested, 2D cell culture showed rather dim signals for the four repetitive dots, while ULA 3D (spheroids formed using ultra-low attachment well plate) showed a strong signal, with similar intensity as our method, which supported the hypothesis that dispase does not affect cell-cell junctions. What is more, dispase is not inhibited by serum, so it can work together with serum media. After lifting off, the cell sheets thickened vertically and shrunk horizontally. Due to this, the cells were no longer subjected the cell-dish tension and were allowed to assume a much rounder morphology when compared to the flatter morphology typically observed in petri dishes. During the shaking process, dispase always acts as spheroid-spheroid connection inhibitor, and working together with the shear force, the spheroids can be always separated without aggregation. As significantly large size (usually average diameter > 200 μm) are formed, spheroids would expose to very large shear force, and so that it is impossible to form stable spheroid-spheroid connection even without dispase. Therefore, dispase is not necessary in this case.

The speed of shaking was also identified to affect spheroid formation. Even though the cell-ECM and cell-petri-dish connections were inhibited by dispase, the cell-cell connection affinity was still retained. At a lower speed (30 rpm), aggregations began to form, but when the shaking speed was higher than 60 rpm, aggregation barely occurred and the spheroid growth showed little difference at different shaking speeds. However, since the circular petri dishes were under orbital shaking, secondary flow (Fig. S3) would induce spheroid collection in the center of petri dishes, especially when the shaking speed was higher and spheroids were bigger. This would cause insufficient nutrition and metabolite exchange and in some extreme cases that the spheroids would be continuously expose to air. Therefore, a proper shaking speed is required to form and maintain robust spheroids.

Control experiments also show the importance of both shaking and dispase on tumor spheroids formation. As shown in Fig. S1A, when cell sheets were shaken in dispase free media, they tended to stack on top of each other, and form large aggregations. There is more and more evidence showing that tumor cells also produce ECM^22,23^, and the possible reason for this phenomenon is cells on one sheet have a rather high affinity to ECM formed on another sheet in dispase free media. While in dispase doped media, the affinity was inhibited, and cell sheets could curl separately and form spheroids. Dispase also softened cell sheets, making them much easier to curl. Other research also supports that retaining cell-cell contact enables tumor spheroid formation^18^. Thick cell sheets with multiple cell layers were not soft enough to allow curling, even with dispase introduced, and the cell junctions were still sufficiently strong to keep the sheet shape, resulting in large, flat, cookie-like structures, as shown in Fig. S1C. On the other hand, stopping the shaking allowed cell junction formation between spheroids, so they formed large cell aggregates, as shown in Fig. S1B, even though cell-ECM connections were inhibited by dispase. However, after significantly large spheroids were formed, the requirement for dispase to inhibited cell-ECM connections became unnecessary, because the contact area between spheroids was much smaller than between cell sheets, leading to a lower possibility that spheroids would bind to each other under similar shear flow. Even though it has negligible negative effect on cell and spheroids growth (Fig. S2), there is no need to keep dispase in media when significantly large spheroids were formed.

The size of the spheroids produced by this method were not uniform (Fig. 4A,C) because the original size of cell sheets were hard to control; furthermore, cell clusters falling off from large spheroids could initiate new smaller spheroids. However, as discussed in the Introduction, the uniformity of spheroids is a key factor that influences their application. Sieve, the ancient but practical and easy tool, is used to separate spheroids with large size distribution, and result in a series of narrow size distribution spheroids. The diameter of filtered spheroids was larger than the mesh size of the larger sieve used for filtration. This was due to spheroids which were soft and flexible allowing them to squeeze through the meshes slightly smaller than themselves. What was more, the mesh pores were square in shape (Fig. S5D), which resulted in even smaller resistance for larger spheroids passing through. This also explains the relatively larger size distribution of spheroids compared to the sizes of meshes that were used. The uniformity can be improved by using stainless steel meshes with smaller incremental steps in pore size.

Table 1 shows the advantages and disadvantages of our method and other popular methods on tumor spheroid formation. Depending on the initial cell seeding concentration and culturing time, both Liquid-Overlay and Hanging-Drop methods showed advantages on uniformity and size controllability. However, each spheroid formed in a droplet or in a well, which lead to low efficiency on quantity, showing a remarkable drawback on applications that needed large amount of spheroids, such as drug screening. These two methods also do not allow for dead cell removal, which might have negative interference on some applications. The spheroid growth is even more restricted in the Hanging-Drop method, due to it being nearly impossible to change media. There are commercially available devices that can be used for tumor spheroids formation based on the principle of Liquid-Overlay and Hanging-Drop methods, such as the Corning^®^ Costar^®^ Ultra-Low Attachment 96 Well Plate and the Kuraray^®^ ‘Multiple Pore Type’ device. The basic principle of the Corning ultra-low attachment well plate is the same as the Liquid Overlay method, and it is one of the most standard methods for tumor spheroids generation nowadays. The Liquid Overlay method was performed in the well plate and the equivalent diameter (range, mean ± SD, CV, n) of the obtained spheroids are (275–350 µm, 312 ± 23 µm, 7.37, 32), respectively, which was similar to our results. However, our method still shows remarkable advantages such as high quantity, media refresh, dead cells removal, and cost reduction over Liquid Overlay method. The basic principle of the ‘Multiple Pore Type’ device by Kuraray is similar to the Hanging Drop method, but this device shows higher productivity and convenience. However, since the initial seeding is introduced at once to the whole chamber of the device, the number of cells that fall into each hole would vary, leading to a large variance in the formed spheroids size. As the results show on the Kuraray website, the size of spheroids formed with the devices was more widely dispersed compared to our method (Fig. S7).

**Table 1:** Advantages and disadvantages of our tumor spheroid formation method and other popular methods.

	Advantages	Disadvantages
Liquid-Overlay^8^	Uniformity Controllable size Media refresh	Low quantity No dead cells removal
Hanging-Drop^3^	Uniformity Controllable Size	Low quantity No media refresh No dead cells removal
Magnetic Levitation^11^	High quantity Media refresh	Low uniformity Uncontrollable size Introduce magnetic particles
NASA Bioreactor^13^	High quantity Media refresh	Bioreactor needed Low uniformity No dead cells removal
**This method**	**High quantity** **High uniformity** **Controllable Size** **Media refresh** **Dead cells removal** **Facile** **Inexpensive**	**Relative long time on cell sheet growth** **Manual steps on size uniformization**

It is easy to produce large amount of spheroids using the Magnetic levitation and NASA Bioreactor methods, and media exchange is much easier to realize, however the uniformity and size controllability are not attainable. Our method, on the other hand, basically gathered most of the advantages and avoided most of the disadvantages mentioned: the average size of spheroids can be controlled by tuning the cell sheets culturing period and spheroid shaking period; the uniformity can be controlled by a set of sieves which were home-made by stainless steel meshes; thousands of spheroids can be produced from rather limited initial cell populations within 2 weeks without expensive consumables and sophisticated instruments and skills; frequent media exchange ensures robust spheroid growth; and dead cells and debris can be easily removed. For cost effectiveness, as an example, our method also shows an extreme advantage over the Liquid-overlay method. If only considering the extra costs for making these spheroids (costs beyond tissue culture), our method only needs dispase solution and after calculation the maximum cost for a single spheroid is only $0.00275. In contrary, the Liquid-overlay method needs $0.149 for a spheroid in a well (Corning^®^ Costar^®^ Ultra-Low Attachment 96 Well Plate). In the calculation, the 800 cell/dish original seeding density was assumed to yield 200 desired size spheroids (which is a minimum estimation, because usually much more spheroids with desired size can be obtained), and only 1 mL dispase solution was needed. In fact, for smaller spheroids, 2000 cell/dish seeding density was also tried and can easily result in > 1000 spheroids of desired size. A few quantitative comparisons were made in Table 2, in which most data were from^15^. Our method showed obvious advantages over other methods. It may find applications in anti-cancer drug screening, *in vitro* tumor studies, and may also be applied to primary cellular spheroid formation or even multi-cell type spheroids and organoid formation.

**Table 2:** Quantitative comparison between our method and other methods. The data of the three methods except Liquid Overlay were from^15^.

	^*^Time Required (day)	^*^No. Cell Required (×10^6^)	Equivalent Diameter (µm) (range, mean ± SD, CV, n)	Amount of Harvested Spheroids	▼Ratio of Spherical Spheroids (SI ≥ 0.9)
^&^Liquid Overlay	7	3	275–350, 312 ± 23, 7.37, 32	A well a spheroid	High
Hanging-Drop	7	0.6	200–500, 359 ± 95, 26.5, 38	A drop a spheroid	Low
Magnetic Levitation	7	0.5	200–500, 347 ± 87, 25.1, 28	No Limit	Low
NASA Bioreactor	15	40	500–1100, 897 ± 98, 11.0, 192	No Limit	Low
**This method**	^#^ **9–15**	**0.0001–0.001**	^▲^ **172–241, 201 ± 13, 6.35, 460**	**No Limit**	**Very High**

^*^Time and number of cells needed to obtain sufficient spheroids to fill a 96-well plate;

^&^Liquid Overlay was performed by using the Corning^®^ Costar^®^ Ultra-Low Attachment Multiple Well Plate, and the initial seeding density was 3000/well. A centrifuge was performed after cell seeding to ensure a high level of sphericity. Without centrifuge, spheroids would have low ratio of SI ≥ 0.9.

^#^Depending on which size spheroid was needed, bigger ones needed more time;

^▲^The data was obtained from 460 spheroids filtered by 120 and 144 µm sieves;

^▼^Referring to^28^, the SI = Sphericity index was calculated from ${SI}=\frac{{\rm{\pi }}\sqrt{\frac{4{\rm{A}}}{{\rm{\pi }}}}}{{\rm{P}}}$, where A and P was the projected area and perimeter of spheroids obtained from ImageJ; the case that >90% spheroids were spherical (SI ≥ 0.9) was considered ‘very high’, >50% was considered ‘high’, and <50% was considered ‘low’.

## Experimental

### Materials and Cell Lines

Cells were cultured in Dulbecco’s modified Eagle’s medium (DMEM, Life Technology), supplemented with 10% fetal bovine serum (FBS, Invitrogen) and 1% Antibiotic & Antimycotic (ThermoFisher). Fluorescence dyes (Calcien AM, and Propidium Iodide (PI)) were purchased from ThermoFisher. Dispase II was purchased from EMD Millipore. Doxorubicin and Paclitaxel were purchased from LC labs. p-nitrophenyl phosphate (PNPP), 1-Step™ PNPP Substrate Solution was obtained from ThermoFisher. All chemicals that are not mentioned are directly used without purifying.

The human colorectal cancer cell line, HCT-116, (purchased from ATCC) was used as model tumor cells in this work. Cultured in 5% CO_2_ and at 37 °C, HCT-116 cells were incubated with DMEM. The medium was changed every 2 days. When the cells reached 80–90% confluency, they were subcultured and 0.05% Trypsin −0.53 mM EDTA was used for cell detachment. A431 (human epidermoid carcinoma cell line) and HeLa (human cervical carcinoma cell line), which were cultured under same conditions, were kindly provided by Dr. Damien Thevenin from the Department of Chemistry at Lehigh University. U87 MG cell line (human primary glioblastoma cell line) was purchased from ATCC.

### MCTS Formation and Size Uniformization

At 70–90% confluency, the cells were trypsinized, centrifuged, and seeded at low concentrations (usually 800 cells per dish) in 6-cm tissue-culture treated dishes for a range of 5 to 14 days, to ensure a spare distribution. The cell density that was seeded onto petri dishes was less than 40/cm^2^. When a desired cell sheet size was reached the media was aspirated, and after washing with PBS, the cells were treated with 500 μL of dispase for 20 minutes in the incubator. Medium was then added in addition to the dispase, at a 1:6 ratio of dispase to media. These dishes were then placed in the incubator on an orbital shaker at 60 rpm. Usually spheroid formation could be observed after 72-hours and the media without dispase was changed every 48 hours using home-made sieves (Illustration could be found in Fig. S5) to refresh media as well as to remove dead cells and small debris. A full set of the sieves was used for spheroid size uniformization. Spheroids with a narrow distribution of size could be obtained using two sieves of adjacent sizes. Specifically as an example, spheroids which passed through the 500 µm sieve but did not pass through the 400 µm sieve were considered to be spheroids of sizes from 400–500 µm.

### MCTS Characterization

Basic phase contrast microscope images were obtained from Olympus IX70. Cell viability was measured via dead/live staining dye (Calcein-AM to indicate the live cells, and PI to identify dead cells, diluted in PBS at 5 μM and 10 μM respectively) and observed by the same microscope. Spheroid histology was documented in spheroids of different size ranges following routine fixation, paraffin-embedding procedures, sectioning (5 µm), and H&E (hematoxylin/eosin) staining, which were performed by Histowiz (New York, USA).

3D structure of tumor spheroids was obtained by optical coherence tomography (OCT), see Fig. S8 and ref.^24–26^ for more information about this modality. Details of the configuration of the spectral domain OCT employed in 3D tumor spheroid imaging can be found in ref.^27^. An individual tumor spheroid was placed in a petri-dish under the objective of the OCT system. A 3D dataset with 400 × 400 × 1024 pixels in each dimension was obtained by OCT, which corresponded to an actual volume of 2.0 × 1.7 × 2.2 mm^3^ in XYZ directions. The total acquisition time for each dataset was ~10 s. A custom post-processing procedure was employed on the obtained OCT dataset to retrieve 3D OCT structure images of the spheroid (Fig. S8,B,C,F,G). Next, 3D rendering of the tumor spheroid can be generated from OCT structural images by Amira (Fig. S8,D,H).

Tumor spheroid vitality was characterized by a modified acid phosphatase (APH) assay, which was introduced in^18^. In brief, spheroids were placed into ultra-low attachment well-plate individually, and the well-plates were then centrifuged at 1500 rpm for 10 min using a NuAire CF 48-R centrifuge. Supernatant was removed carefully, and 200 μL PBS was injected to wash away the remaining media. Centrifugation was repeated, and 100 μL PNPP was added, followed by a 90-min incubation at 37 °C. 10 μL 2 N NaOH was used to stop the reaction and absorbance readings at 405 nm were recorded on a TECAN Infinite F200 well plate reader.

### Drug Test

The therapeutic efficacy of doxorubicin (Dox) and Paclitaxel (Pac) were evaluated in 3D spheroid-based assays. Uniformly sized spheroids of each cell line were chosen for treatment with a monolayer serving as control. Individual tumor spheroids (diameter ~ 400 μm) were transferred to round-bottomed 96 well plates, and incubated in drug-doped media for 72 hours with the following concentrations: 1 nM, 3 nM, 10 nM, 20 nM, 50 nM, 100 nM, 200 nM, 500 nM, and 1000 nM in standard medium. Microscope images of the original tumor spheroids were captured, as well as images after drug treatment. APH assay was performed to determine the viability. The data analysis was processed using OriginLab 8.5.

## Electronic supplementary material

Supplementary Information
