# Supplementary material for: Facile Tumor Spheroids Formation in Large Quantity with Controllable Size and High Uniformity
Source: Sci Rep. 2018 May 1;8:6837. doi: 10.1038/s41598-018-25203-3 (PMC5931581; doi:10.1038/s41598-018-25203-3)
Supplement: Supplementary file 1 — Supplementary Information [file 41598_2018_25203_MOESM1_ESM.docx]

**Supplementary Information**

**Facile Tumor Spheroids Formation in Large Quantity with Controllable Size and** **High Uniformity**

**Wentao Shi,^1^ Jean Kwon,^1^ Yongyang Huang,^2^ Jifu Tan,^3^ Christopher G. Uhl,^1^ Ran He,^4^ Chao Zhou,^1,2^ Yaling Liu*^,1,4^**

^1^Department of Bioengineering, Lehigh University, Bethlehem, Pennsylvania 18015, USA

^2^Department of Electrical and Computer Engineering, Lehigh University, Bethlehem, Pennsylvania 18015, USA

^3^Department of Mechanical Engineering, Northern Illinois University, DeKalb, IL 60115, USA

^4^Department of Mechanical Engineering and Mechanics, Lehigh University, Bethlehem, Pennsylvania 18015, USA

^*^Corresponding author E-mail:[yal310@lehigh.edu](mailto:yal310@lehigh.edu)


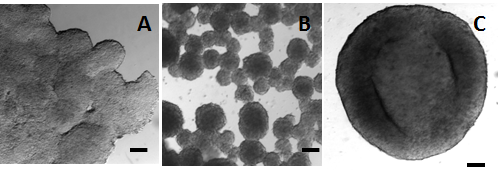


Fig. S1. A few control experiments for tumor spheroids formation. (A) Controlled by using dispase-free media when shaking, the image was taken at shaking day 1; (B) Controlled by stopping shaking for 24h at day 7; and (C) Controlled by using large cell sheet (Sheet growth = 15 days), the image was taken at shaking day 5. All scale bars are 200 µm.


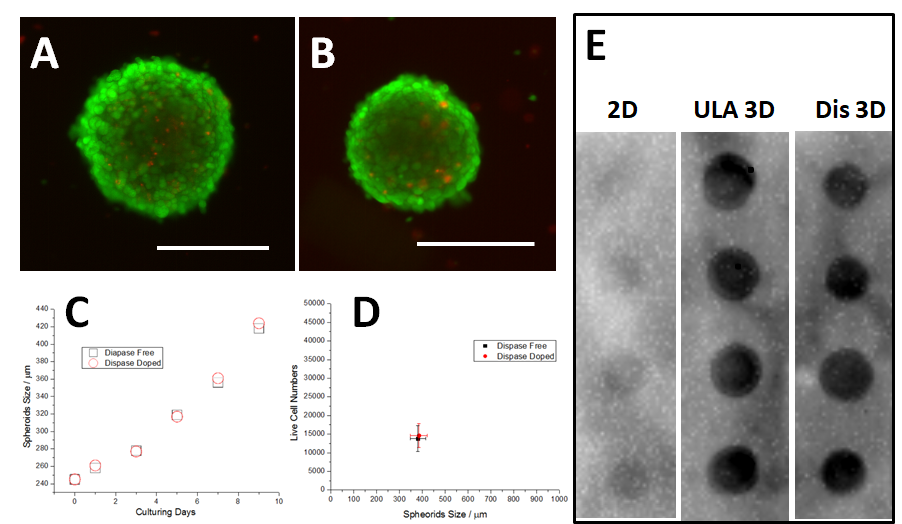


Fig. S2. Dead/Live staining fluorescence images for two typical HCT-116 spheroids shaken with (A) and without (B) dispase. The size change of the two comparable spheroids were also recorded for 10 days, and the results are shown in (C), indicating that there was negligible difference between with and without dispase treatment. APH experiments (see next part) were also performed to prove the argument, and results are shown in (D). Only a single size of spheroid was studied (around 400 µm), and at least 12 spheroids were used for each point in (D). (E) Dot plot results show that the cell to cell junction protein E-cadherin was not affected by continued dispase treatment. Scale bars = 200 µm.

Dispase was found to have negligible negative effects on cell and spheroid growth. HCT-116 spheroids from the same batch were cultured under shaking-conditions in dispase-free and dispase-doped media, and the size change was monitored for 10 days. As the fluorescence images shown in Fig. S2- A and B, spheroids do not show a remarkable difference between shaking-conditions in dispase-free and dispase-doped media in dead/Live staining, with most of surface cells alive. The average size change over 10 days of culturing in the two situations also does not show remarkable difference (Fig. S2-C), indicating a negligible negative effect of dispase on cell and spheroid growth. Additional evidence of live cell numbers in spheroids obtained from the two situations did not show remarkable difference (Fig. S2-D). Dot plot results show that the cell to cell junction protein E-cadherin was not affected by continued dispase treatment (Fig. S2-E). Cell amounts were attempted to maintain consistent for the Dot plot. Same size and same amount of spheroids were used for the ultra-low attachment well plate spheroids (ULA 3D) culture and our method using dispase treatment (Dis 3D), and the amount of 2D cells was used based on the calibration curve in Fig. 5-D.


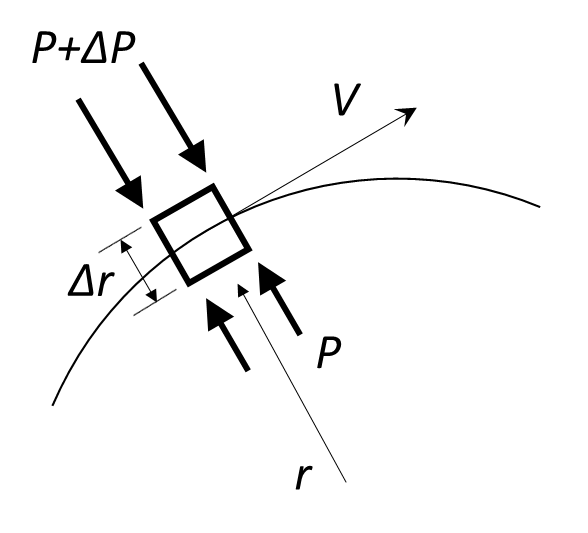


Fig. S3. Secondary flow induced Spheroid collection at the center of the petri dish under orbital shaking.

When the petri dish is under conditions of orbital shaking, it creates a primary spinning flow in the petri dish. A centripetal force toward the center is needed to maintain the spinning motion of the fluid. However, the fluid flow velocity near the boundary wall or at the bottom surface is much smaller than the spinning speed due to the wall friction and viscous force in the boundary layers. To analyze the motion of the bulk fluid, e.g., not in the boundary layer, the forces between shear stress, gravity, pressure forces and momentum should be balanced. The shear stress is negligibly small compared with the pressure and inertial force. The gravity is perpendicular to the velocity, thus it can be safely ignored. The motion of the fluid element can be approximated by Euler’s equation,

$$\frac{\partial P}{\partial r}=\frac{\rho V^{2}}{r}$$

However, for fluid flow close to the boundary layer, the velocity V is low, thus, the centrifugal acceleration cannot balance the pressure gradient. As a result, a secondary flow is formed with the fluid close to the bottom surface moving toward the center of the petri dish, as observed in the collection of spheroid in the center of the petri dish. This was explained by Albert Einstein [Einstein, Albert. "Die Ursache der Mäanderbildung der Flußläufe und des sogenannten Baerschen Gesetzes." Naturwissenschaften 14.11 (1926): 223-224.]


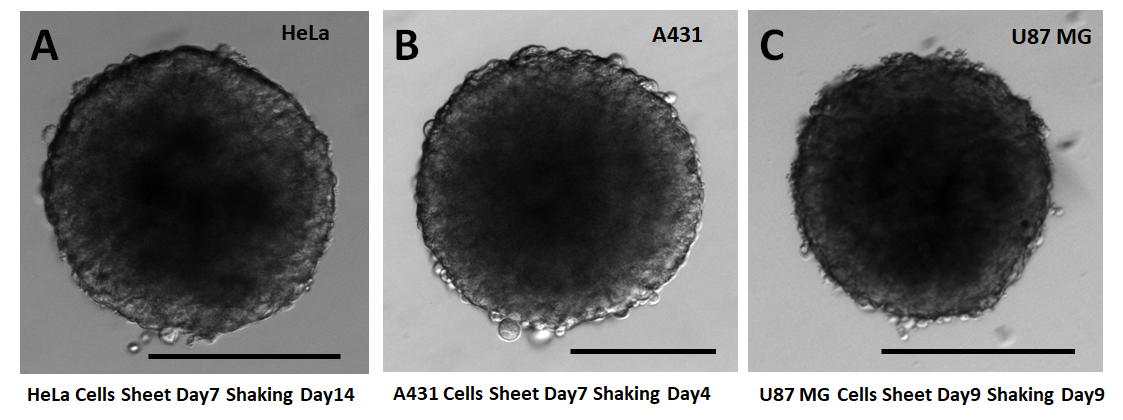


Fig. S4. Other cell lines can also form high quality and high uniformity robust spheroids by using this method. (A) Typical microscope image of HeLa tumor spheroids shaken for 14 days from cell sheet culturing for 7 days; (B) Typical microscope image of A431 tumor spheroids shaken for 4 days from cell sheet culturing for 7 days; and (C) Typical microscope image of U87 MG tumor spheroids shaken for 9 days from cell sheet culturing for 9 days. Scale bars = 200 µm.

Other than HCT116, this method has been successfully used for HeLa (human cervical carcinoma cell line), A431 (human epidermoid carcinoma cell line) and U87 MG (human primary glioblastoma cell line) tumor spheroids formation. HeLa cells grow much slower than HCT116 and A431, but after relatively longer time of culturing, spheroids with desired sizes can still be obtained. Although currently this method is still only used on tumor cell lines, we believe it can be used on primary cells.


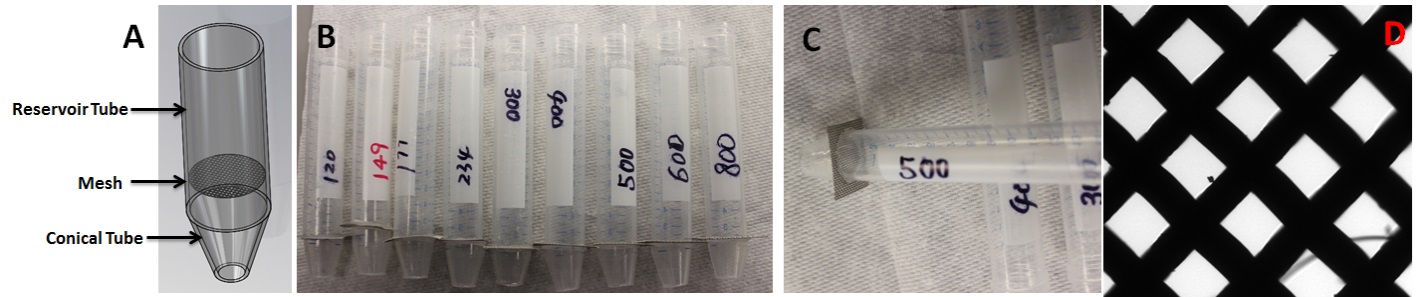


Fig. S5. Illustration (A) and the sample photos (B and C) of the autoclavable sieves for spheroid size uniformization. (D) Typical microscope image of a 149 µm stainless steel mesh, showing square holes with 149 µm side length. Spheroids that are filtered through this type of square mesh could be slightly larger than the mesh size, because they are soft and can squeeze to pass through the mesh.


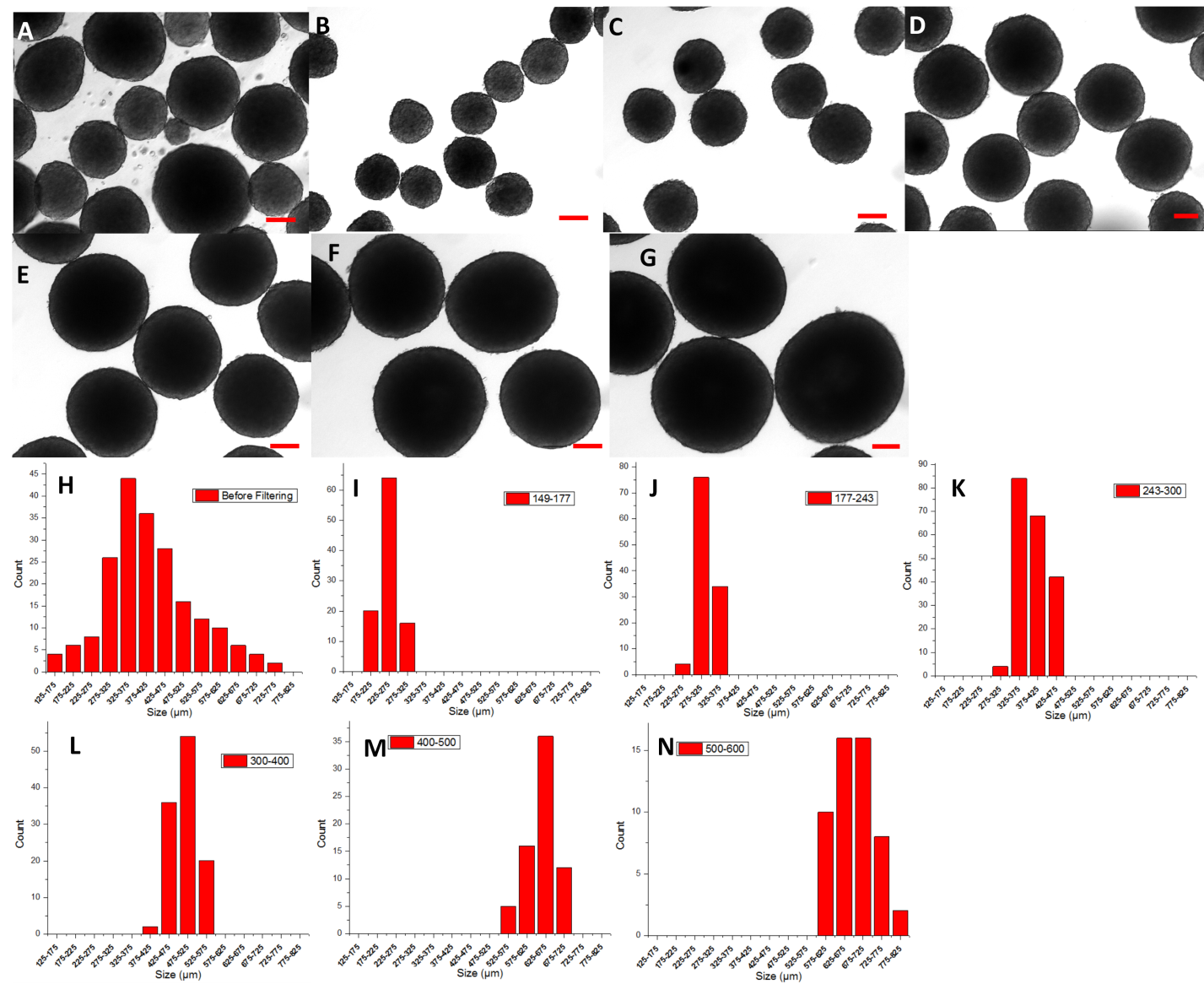


Fig. S6. HCT-116 tumor spheroids size uniformization. (A) A typical image showing the wide size-distributions of the tumor spheroids, taken at shaking day 7 (detached at sheet-growth day 9). (B-G) Tumor spheroids from the same batch as (A) filtered by different sieves. (H-N) Tumor spheroids size distributions before and after filtration, vertically corresponding to (A-G). The numbers such as 149-177 in (I) mean such spheroids passed through the sieve with smaller pore sizes in µm but did not pass the sieve with larger number in µm. Each spheroid count was counted and calculated from 10-30 images taken at certain size distributions. All scale bars are 200 µm.


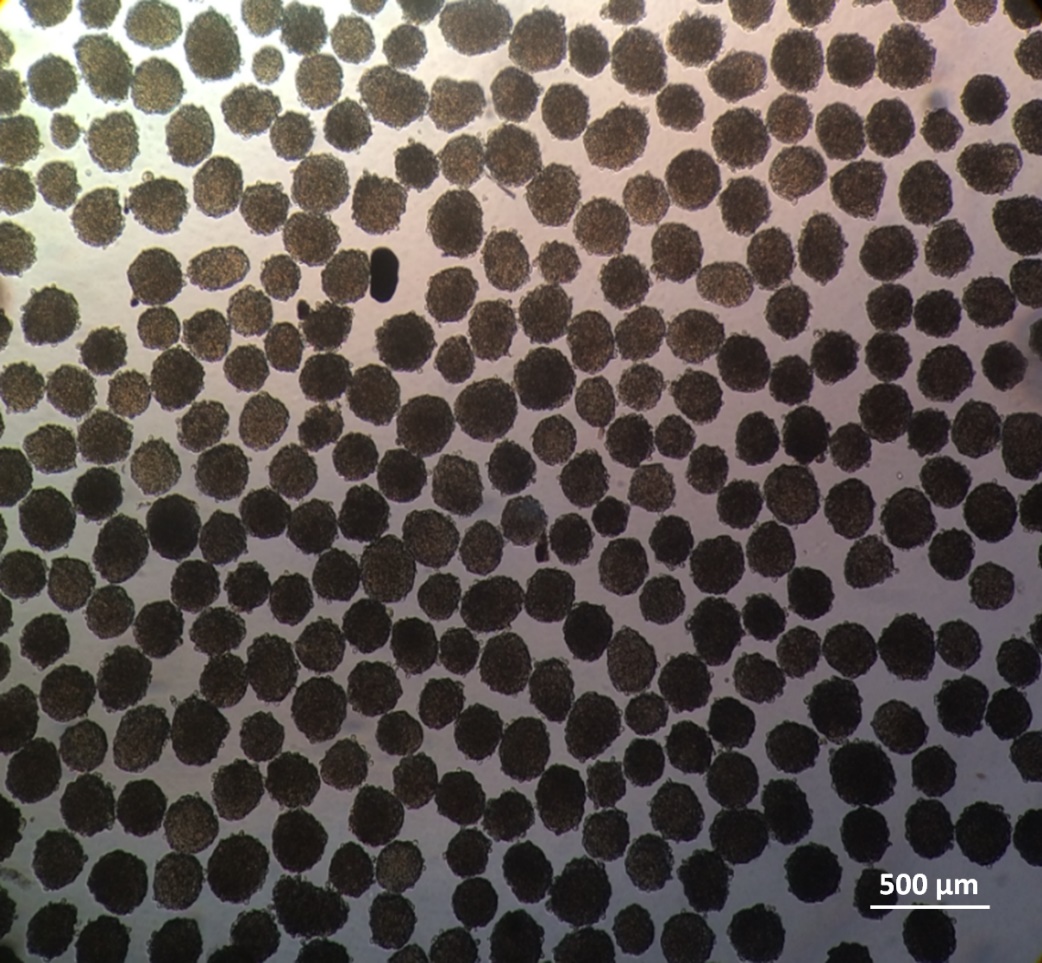

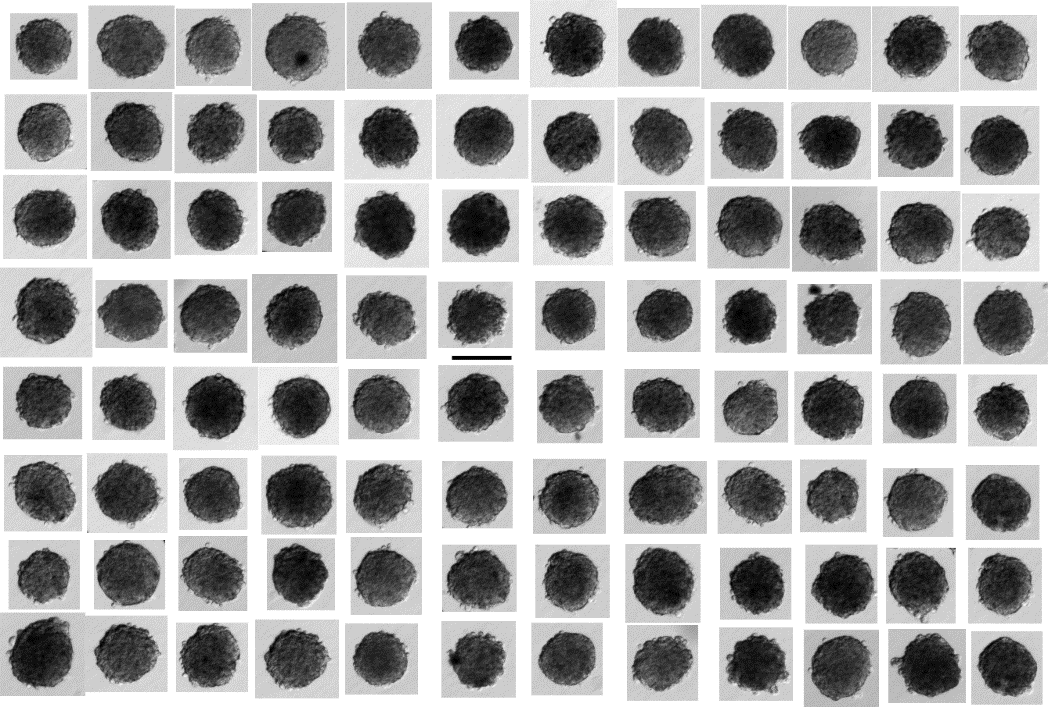


Fig. S7. HCT116 spheroids from 120-144 um sieves. (upper) Low magnification image of highly packed spheroids, and (lower) 96 randomly picked spheroids transferred to 96 well-plate. Scale bar in the middle = 200 µm.


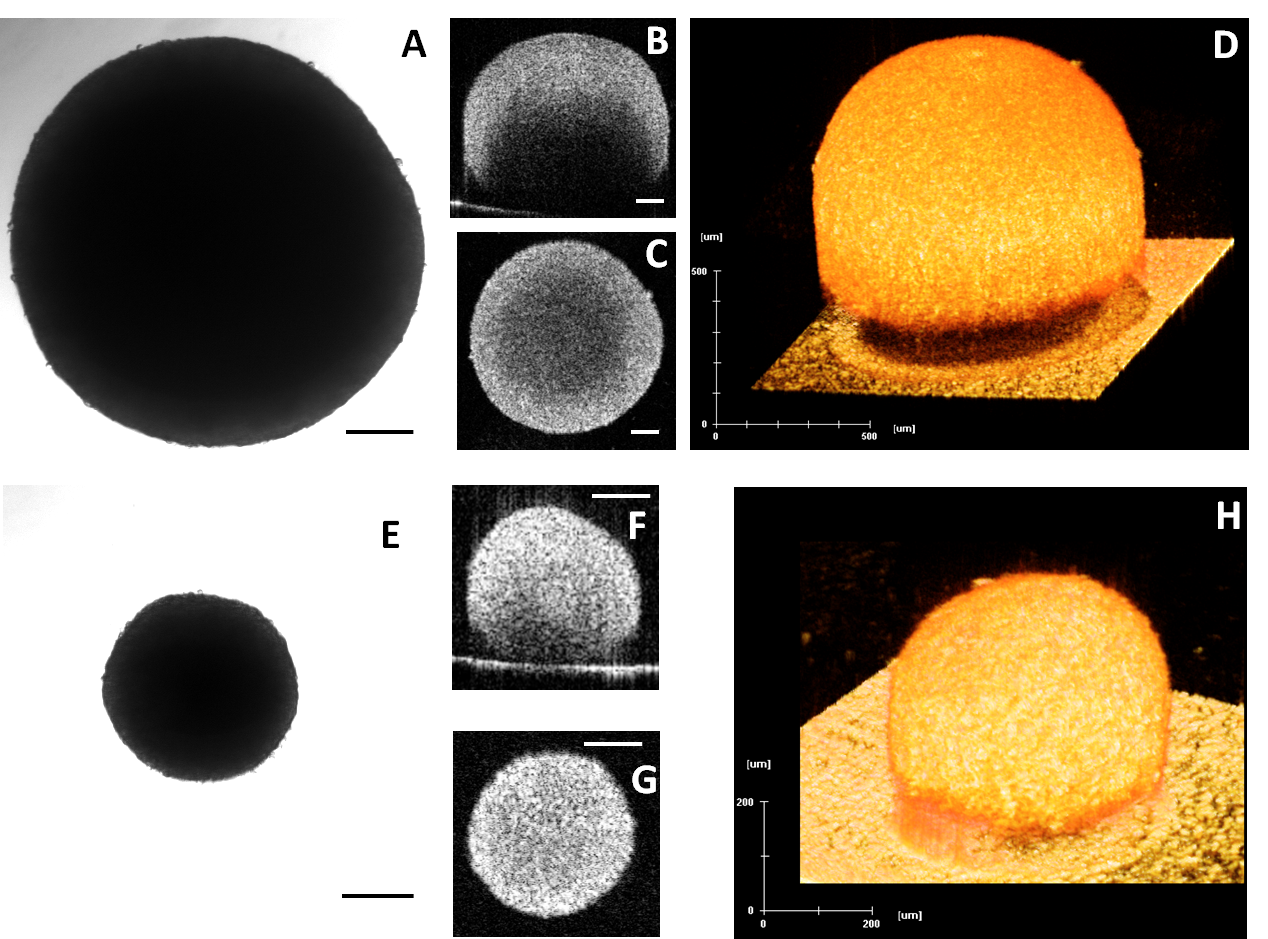


Fig. S8. Two-dimensional (2D) phase contrast microscopic image of a large tumor spheroid with a measured diameter of 1205 µm. B. 2D cross-sectional image of the tumor spheroid obtained by optical coherence tomography (OCT). OCT is able to detect back-scattered light within the tumor spheroid from depths close to 1 mm below the top surface of the spheroid. C. 2D top view image of the tumor spheroid by OCT. D. 3D rendering of the tumor spheroid by OCT to characterize the overall shape and size of the tumor spheroid. E. 2D bright field image of a small tumor spheroid with a measured diameter of 558 µm. F. 2D cross-sectional image of the small tumor spheroid by OCT. G. 2D top view image of the small tumor spheroid by OCT. H. 3D rendering of the small tumor spheroid by OCT. With this culture method, tumor spheroids can retain a spherical shape for a long time with their diameters growing over 1 mm.

Optical coherence tomography (OCT) ^1–3^ is a three-dimensional (3D) biomedical imaging modality based on low-coherence interferometry. OCT detects back-scattered light and reconstructs a 3D image of microstructures within the tissue, with a field of view close to a standard bright field microscope and light penetration of up to 1-2 millimeters below the tissue surface. OCT scanning is fast, non-invasive and label-free. Moreover, obtained OCT images have micron-scale resolution in both axial and transverse dimensions ^4^. OCT has its wide applications in the field of ophthalmology and cardiology ^5,6^. Recently, several groups have employed OCT to quantitatively characterize the 3D tumor spheroids ^7,8^.
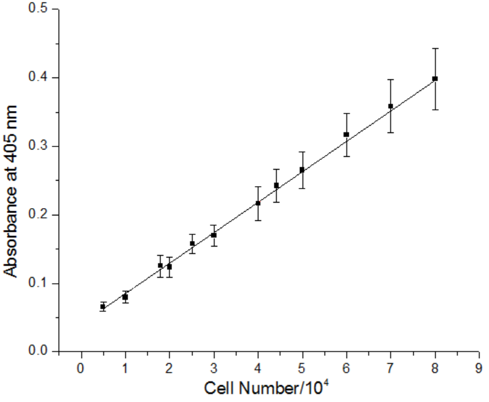


Fig. S9. The relationship between live cell number of single cell suspension in a well of 96 well-plate and its absorbance at 405 nm on a well-plate reader.


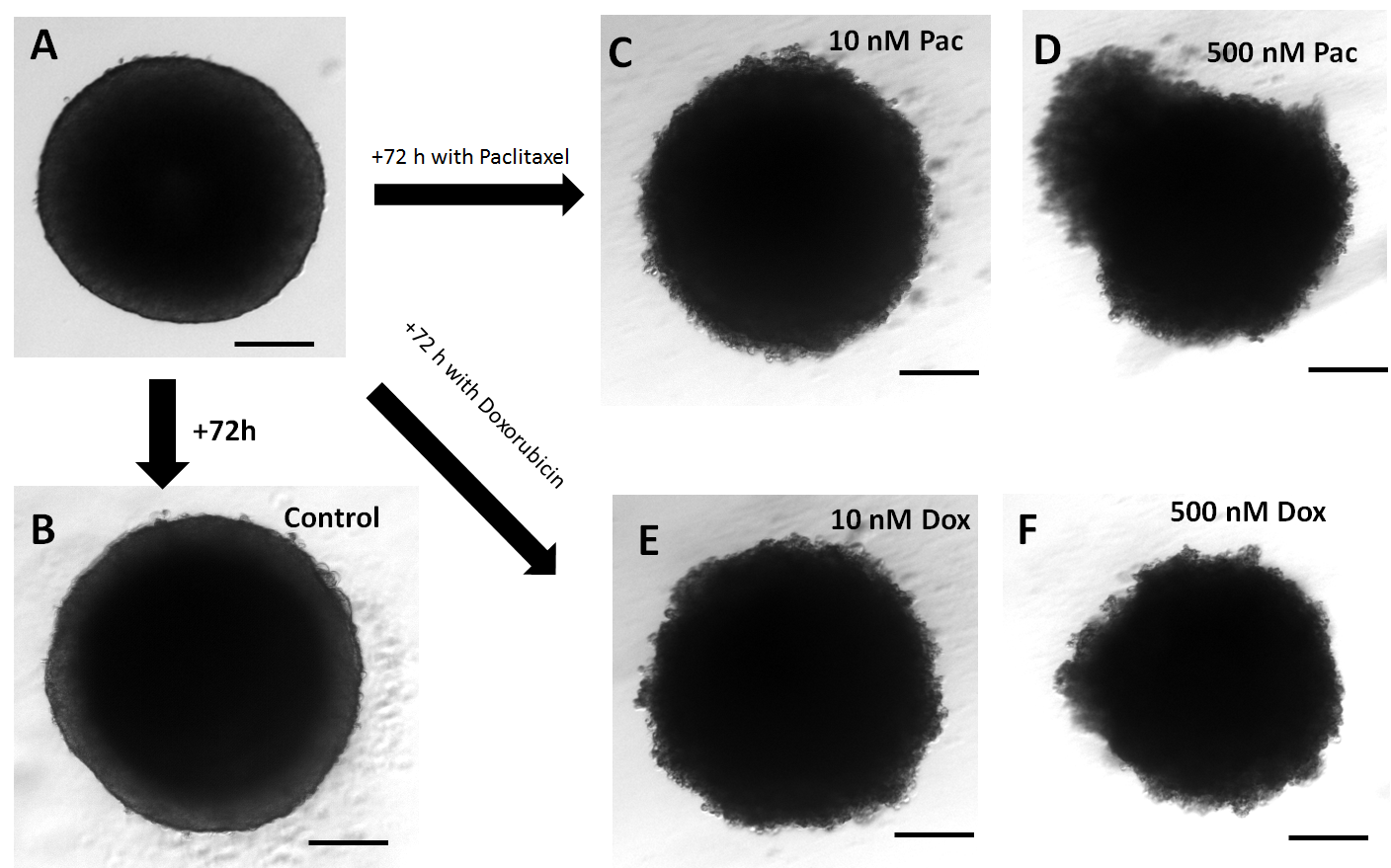


Fig. S10. Spheroid integrity after treatment with Paclitaxel and Doxorubicin. Representative phase contrast images of the original spheroids (A), controlled spheroids after 72 h non-drug treatment (B), spheroids with 10 nM (C) and 500 nM (D) Paclitaxel for 72 h, and spheroids with 10 nM (E) and 500 nM (F) Doxorubicin for 72 h. Similar size spheroids (diameter ~500 µm) were treated with different concentrations of Paclitaxel and Doxorubicin for 72 h, and (A) shows a typical controlled spheroid, while (B) shows a spheroid that was not treated by any drug for 72 h.


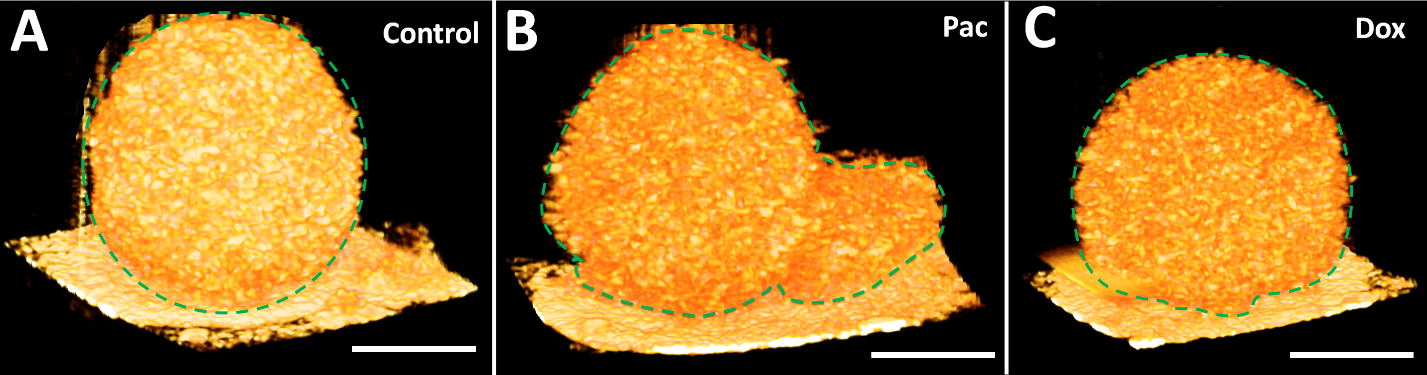


Fig. S11. 3D rendered OCT images of spheroids after drug treatment for 72 h: (A) Control, (B) Paclitaxel (500 nM), and (C) Doxorubicin (500 nM). The initial size of spheroids were about 250 µm.

**References:**

1 T. Klein and R. Huber, *Biomed. Opt. Express*, 2017, **8**, 828.

2 J. Fujimoto, E. Swanson, Vermeulen D, Sattmann H, Chavez-Pirson A and D. W., *Investig. Opthalmology Vis. Sci.*, 2016, **57**, OCT1.

3 D. Huang, E. Swanson, C. Lin, J. Schuman, W. Stinson, W. Chang, M. Hee, T. Flotte, K. Gregory, C. Puliafito and al. et, *Science (80-. ).*

4 I. Grulkowski, J. J. Liu, B. Potsaid, V. Jayaraman, C. D. Lu, J. Jiang, A. E. Cable, J. S. Duker and J. G. Fujimoto, *Biomed. Opt. Express*, 2012, **3**, 2733–51.

5 G. J. Tearney, S. Waxman, M. Shishkov, B. J. Vakoc, M. J. Suter, M. I. Freilich, A. E. Desjardins, W.-Y. Oh, L. A. Bartlett, M. Rosenberg and B. E. Bouma, *JACC. Cardiovasc. Imaging*, 2008, **1**, 752–61.

6 L. Liu, J. A. Gardecki, S. K. Nadkarni, J. D. Toussaint, Y. Yagi, B. E. Bouma and G. J. Tearney, *Nat. Med.*, 2011, **17**, 1010–1014.

7 M. Sharma, Y. Verma, K. D. Rao, R. Nair and P. K. Gupta, *Biotechnol. Lett.*, 2007, **29**, 273–278.

8 O. J. Klein, Y. K. Jung and C. L. Evans, *Methods*, 2014, **66**, 299–311.
